# Multimodal MRI Brain Tumor Image Segmentation Using Sparse Subspace Clustering Algorithm

**DOI:** 10.1155/2020/8620403

**Published:** 2020-07-04

**Authors:** Li Liu, Liang Kuang, Yunfeng Ji

**Affiliations:** ^1^School of IoT Engineering, Jiangsu Vocational College of Information Technology, Wuxi 214153, China; ^2^School of Computer and Software, Nanjing University of information Science& Technology, Nanjing 210044, China

## Abstract

Brain tumors are one of the most deadly diseases with a high mortality rate. The shape and size of the tumor are random during the growth process. Brain tumor segmentation is a brain tumor assisted diagnosis technology that separates different brain tumor structures such as edema and active and tumor necrosis tissues from normal brain tissue. Magnetic resonance imaging (MRI) technology has the advantages of no radiation impact on the human body, good imaging effect on structural tissues, and an ability to realize tomographic imaging of any orientation. Therefore, doctors often use MRI brain tumor images to analyze and process brain tumors. In these images, the tumor structure is only characterized by grayscale changes, and the developed images obtained by different equipment and different conditions may also be different. This makes it difficult for traditional image segmentation methods to deal well with the segmentation of brain tumor images. Considering that the traditional single-mode MRI brain tumor images contain incomplete brain tumor information, it is difficult to segment the single-mode brain tumor images to meet clinical needs. In this paper, a sparse subspace clustering (SSC) algorithm is introduced to process the diagnosis of multimodal MRI brain tumor images. In the absence of added noise, the proposed algorithm has better advantages than traditional methods. Compared with the top 15 in the Brats 2015 competition, the accuracy is not much different, being basically stable between 10 and 15. In order to verify the noise resistance of the proposed algorithm, this paper adds 5%, 10%, 15%, and 20% Gaussian noise to the test image. Experimental results show that the proposed algorithm has better noise immunity than a comparable algorithm.

## 1. Introduction

Tumor is one of the common malignant diseases that endanger human health. According to origin, tumors are generally divided into primary and secondary. Compared with breast, lung, and esophageal tumors, the incidence of brain tumors is relatively low. Compared with the overall incidence of human tumors, it accounts for about 1.4%; however, the mortality rate reaches 2.4% of human tumors [1]. Glioma is the most common primary brain tumor in adults. It is mainly distributed in glial cells and the tissues it infiltrates, and it is the most common malignant brain tumor. According to the nature of tumors, gliomas are generally divided into benign and malignant. Benign gliomas generally grow relatively slowly, patients have a longer survival period, and the long course of disease is the main manifestation of benign gliomas. Malignant gliomas generally grow faster, and the short course is a prominent manifestation of malignant gliomas. If the intracranial lesions can be detected as soon as possible, and the corresponding treatments can be implemented, the health hazards of brain tumors to humans can be reduced. CT or MRI imaging to analyze the pathological state of brain tissue is currently the mainstream method for examining brain tumors. Different imaging techniques have different advantages for tumor diagnosis. Compared with CT imaging, MRI uses a noninvasive imaging method, which can provide the observer with high-quality images without damage and skull artifacts, with clear anatomical structure, and with very good soft tissue resolution. At the same time, intracranial images in any direction can be obtained by adjusting the relevant parameters. In addition, using different imaging sequences, MRI of different angles or modalities of the same tissue can be obtained. This type of image is generally referred to as a multimodal MRI image.

The effective diagnosis of brain tumors requires the successful segmentation of tumors in brain images. Based on the results of the segmentation, the doctor can determine the shape, size, and specific location of the tumor. According to the segmentation results of the tumor in the image, a corresponding treatment plan is given. Due to the increase in the number of patients with brain diseases and the development of intelligent diagnostic technology, the research work based on brain tumors continues to increase. The International Conference on Medical Image Computing and Computer-Assisted Intervention (MICCAI) began in 2012 and has organized competitions based on multimodal brain tumor segmentation for four consecutive years, greatly promoting the development of brain tumor segmentation technology. It is of great research value and practical significance to improve the diagnosis efficiency by mining potential pathological information of MRI brain tumor images through image processing technology and machine learning methods. However, the tumor is only characterized by gray-scale information on the MRI image, and the edge of the tumor structure and the normal tissue have significant gray-scale similarity. Simultaneously, the size, location, shape, and corresponding expansion of the tumor in the brain tissue will show different states with different patients. These characteristics pose challenges to the development of tumor segmentation technology.

**Figure 1:**
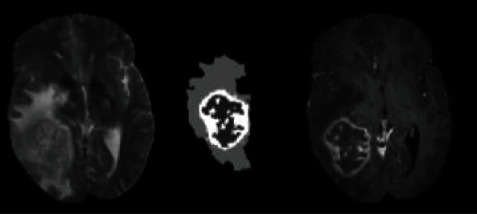
Tumor labels manually segmented on T1c and T2 modal images.

The so-called brain tumor segmentation refers to the process of segmenting various tumor tissues from a variety of conventional brain tissues. In general, the segmentation methods of brain tumor images can be summarized into three categories [2]: purely artificial, semiautomatic, and fully automatic segmentation. Manual segmentation refers to manually drawing the outline of the target tissue.  Figure 1 is a schematic diagram of manual segmentation. Manual segmentation is boring and time consuming, so it cannot meet the growing demand for segmentation. In addition, each segmenter has a different segmentation style, which leads to deviations in segmentation results. Although manual segmentation has many disadvantages, manual segmentation has the highest segmentation accuracy so far, and is often used as the ground truth for automatic segmentation. Semiautomatic segmentation is sensitive to initialization. Users need to input certain initialization data to get the final segmentation result. Fully automatic segmentation does not need to set any parameters manually and can automatically locate and segment the tumor area.

**Figure 2:**
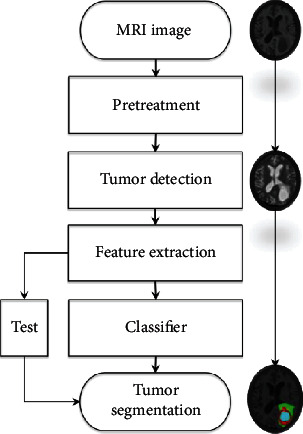
Flow of the brain tumor segmentation method based on the discriminant model.

There is still a lack of a general method that can process all brain tumor images and obtain satisfactory results currently. Usually, the segmentation method is aimed at specific image data. Reviewing related literature, tumor image segmentation methods can be summarized as follows:

1. Threshold based method. The practicability and segmentation effect of this method are very good. The histogram in the global threshold can be expressed as a bimodal model, and a single threshold can be used to distinguish tumor from background. Reference [3] proposes an unsupervised method to enhance pixel grayscale and utilize it to segment brain tumors in T1c images. If there are multiple types of regions in the image, a multithreshold strategy needs to be added to the segmentation method, called local threshold. For the local mean, it can be obtained by estimating the local statistical characteristics, such as gray average [4] and data Gaussian distribution [5]. Generally, the threshold-based method cannot use all the information of the MRI image, and the segmentation result is relatively rough. Therefore, the threshold-based method in brain tumor segmentation is first applied
2. Area-based approach. Through predefined similarity criteria, in the way of merging neighboring pixels in the intersecting areas, the target MRI brain image is divided into the required subareas. Reference [6] applied region growth to MRI tumor segmentation image segmentation with good results. Reference [7] proposed an improved method of region growth. This method obtains a more exact boundary message by reducing the volume effect. The leak gap that may be generated after the division is also filled to a certain extent. As a morphological method, watershed segmentation represents the target contour edge as a partial watershed, which is widely used in brain tumor segmentation. References [8, 9] proposed a multiscale watershed transformation method. Reference [10] constructed an artificially assisted segmentation method by the hierarchical watershed method. From the principle of the watershed segmentation method, this kind of image edge and region watershed conversion easily produces oversegmentation. In order to solve this problem, some related processing methods have been excavated one after another
3. Pixel classification method. The collected MRI brain tumor data generally has two formats, namely, 2D slices and 3D volume. If it is a brain tumor segmentation based on slice format, its essence is the same as traditional image segmentation. The pixel-based method mainly uses the pixel characteristics of the image, and uses some related classifiers to classify all the pixels in the brain tissue image, so as to achieve the effect of segmentation. Unsupervised classification is mainly represented by clustering [11, 12]. The core idea is to measure the relationship between tumor tissue and other tissues in the tumor image. The supervised classifier [13, 14] mainly uses those labeled training samples to train the relevant parameters in the model, which has reached the optimal tumor segmentation effect [15].
4. Model-based method. Model-based tumor segmentation methods are mainly 3D-oriented volume data, followed by 2D slice data. The most typical are the active contour model [16] and the level set method [17]. On the basis of these two models, tumor segmentation has formed two schools: the segmentation methods based on the generative algorithm and the discriminant algorithm. The generation algorithm uses the unique information of various organizations to predict the information of brain tissue that cannot be captured in the image [18–20]. In some generative models, in order to solve the problem of difficult coding of a priori knowledge of tumors, the diseased tissue of the tumor can be modeled as the desired shape [21–23], or it can be inferred using the given patient image and the tumor growth model's possible location of the tumor structure [24]. Discriminant methods generally require a certain size of training samples [25–27]. After many trainings, the processing effect of the discriminant model is more robust to the effects of MRI image artifacts and grayscale information.  Figure 2 shows the basic flow of model-based tumor segmentation. For effective training, the first step of this type of method is generally to extract local gray-scale differences [28] or gray-scale distribution and other voxel-wise features [29], and then send these features to the discriminant classifier of the model. In order to combine the advantages of discriminant models and generative models, a method called generative discriminant model [30, 31] was proposed

In this paper, the BRATS 2015 competition database is used as the experimental object, and the traditional segmentation method and the sparse subspace clustering method based on sparse representation are used to segment the brain tumor images. The main innovations of this article are as follows:

1. Introduce the sparse subspace clustering algorithm to achieve brain tumor image segmentation. The advantage of this algorithm is to use low-dimensional data to recover and approximate high-dimensional data, effectively reducing the dimension of high-dimensional data while retaining the correlation between the data. The introduction of this algorithm can solve the problem of excessive data dimension
2. This article focuses on the segmentation of MRI brain tumor images under multimodality. In the single-modality image fusion strategy, a simple and fast linear fusion strategy is selected. Before segmenting multimodal images, the image is preprocessed by superpixel segmentation, feature vectors are extracted, and the data dimension is reduced. Experimental results show that for brain tumor segmentation, multimodal brain tumor information can be used as much as possible to obtain more accurate segmentation results

## 2. Related Information

### 2.1. Multimodal MRI Brain Tumor Image Introduction

**Figure 3:**
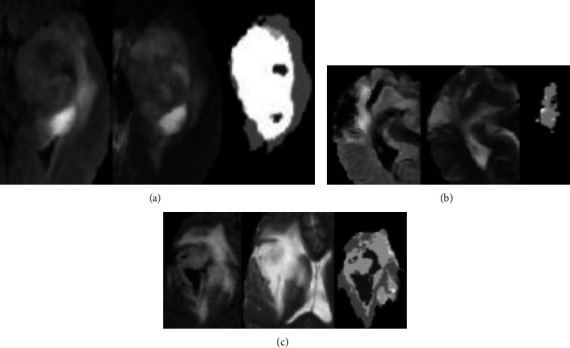
Flair and T2 images and corresponding tumor labels.

Multimodal MRI images are images of the same tissue under different contrasts obtained through different MR development sequences. When tumors and other lesions occur in brain tissue, water molecules existing in free form in brain tumors begin to undergo lesion reactions, such as tissue edema. In Flair and T2 images, the water molecules in the bound state are displayed in the form of high signals. Therefore, it is theoretically feasible to use Flair modal MRI images as the main basis for segmenting the entire tumor. However, due to some special circumstances, the tumor will also show irregular changes in the Flair image. At this time, the image data of the T2 mode can provide additional reference.  Figure 3 depicts three different sets of Flair and T2 images. Among them is (1) the Flair image, (2) the T2 image, and (3) the artificially labeled tumor structure image. The data used in this paper are all from the BRATS 2015 [32] database, and the database includes images in four modes: T1, T1c, T2, and Flair.

### 2.2. Difficulties in MRI Brain Tumor Image Segmentation

There are many difficulties in the segmentation of MRI brain tumor images. These difficulties can be summarized as follows:

1. The most typical problem of MRI comes from the different nonstandard intensity ranges obtained by different scanners. Because of different magnetic field strengths and acquisition protocols, for the same patient, the brain MRI strength values are also different between hospitals
2. The brain tumor itself has no fixed shape or prior knowledge. Brain pathology can appear anywhere in the brain and can have any shape. In addition, the gray value range of this pathology may overlap with the gray value range of healthy tissues, making segmentation of brain tumors more complicated
3. MRI has nonnegligible white Rician noise during the acquisition process [33]
4. Uniform organization is often affected by changes in the spatial intensity of each dimension. This is caused by the bias field effect. The MRI bias affects the smoothed low-frequency signal of the image intensity. This problem requires an offset field correction preprocessing step, which usually increases the intensity value around the brain
5. Large tumors or lesions in the brain may distort the overall structure of the brain, making some procedures impossible to perform. For example, a larger tumor may affect the overall symmetry of the brain, making it impossible to calculate the left-right symmetry feature. In addition, brains with large tumors are difficult to register with healthy brain templates

## 3. Brain Tumor Image Segmentation Based on Sparse Subspace Clustering Algorithm

Sparse representations are widely used in image segmentation algorithms. Sparse representations can effectively reduce the complexity of data operations and bring convenience to the subsequent processing of data. SSC is a clustering algorithm based on sparse representation and subspace clustering [34]. Before segmentation, the target image needs to be preprocessed.

### 3.1. Image Preprocessing

**Figure 4:**
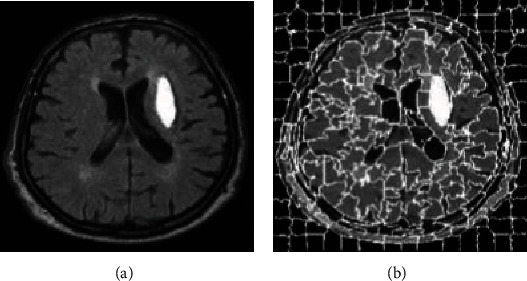
SLIC super pixel segmentation. (a) Original image. (b) SLIC superpixel segmentation image.

Before SSC splits an image, the image needs to be split into superpixels. Superpixels are irregular image blocks composed of a series of adjacent pixels with similar characteristics, such as texture, color, and brightness. It replaces a large number of pixels with a few superpixels, which effectively reduces the amount of data that expresses the features of the picture, thereby reducing the complexity of image postprocessing. Superpixel segmentation algorithms are currently divided into two types, one is based on graph theory, and the other is based on gradient descent, such as Simple Linear Iterative Clustering (SLIC) [35]. The segmentation method based on gradient descent belongs to an iterative segmentation method. First, an initial clustering is given, and then the gradient clustering method is used to modify the result of the previous clustering, and iterate continuously until the convergence condition is satisfied. The superpixel rendering using SLIC segmentation is shown in Figure 4.

### 3.2. Basic Model

**Figure 5:**
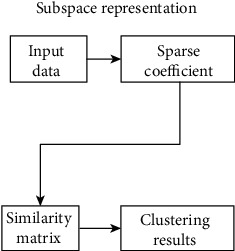
The basic framework of sparse subspace clustering.

The algorithm is to assume that the data is composed of high-dimensional spatial data, and each data can be represented in a low-dimensional subspace. That is, by letting the data in the high-dimensional space be expressed linearly with the data in the low-dimensional subspace, the low-dimensional subspace to which the data belongs can be clearly known, which is beneficial to the clustering operation. The basic framework of sparse subspace clustering is shown in Figure 5.

The SSC model building process is as follows.

Given a set of datasets *X* = {*x*_1_, *x*_2_, *x*_3_, ⋯, *x*_*n*_}, the dimension is *D*, located in *n* linear subspaces {*S*_*i*_},  *i* = 1, 2 ⋯ , *n*. The dimensions of the linear subspace are {*d*_*i*_},  *i* = 1, 2 ⋯ , *n*. Then define the matrix

(1) $$\begin{array}{c} X=\left\{x_{1},x_{2},x_{3},\cdots ,x_{n}\right\}=\left[X_{1},X_{2},\cdots ,X_{n}\right]\times Ζ, \end{array}$$

where *X*_*i*_ ∈ *R*^*D*×*N*_*i*_^ is a matrix of rank *d*_*i*_ composed of the *i*th subspace data. *Z* is the permutation matrix. Subspace clustering is essentially to obtain the *X*_*i*_ ∈ *R*^*D*×*N*_*i*_^ matrix.

Subspace representation means that every data in matrix *X* can be linearly represented by data in the same subspace except for itself:

(2) $$\begin{array}{c} x_{i}={Xa}_{i},\;a_{ii}=0, \end{array}$$

where *a*_*i*_ = [*a*_*i*1_, *a*_*i*2_, ⋯,*a*_in_]^*T*^. Formula (1) can be written in matrix form as follows:

(3) $$\begin{array}{c} X=XA,\;A_{ii}=0, \end{array}$$

where **A** = [*a*_1_, *a*_2_, ⋯, *a*_*n*_] ∈ *R*^*N*∗*N*^ is a sparse matrix. In order to make the sparse matrix **A** the most sparse, that is, the nonzero values in matrix **A** are minimized, by obtaining the *l*_0_− norm to minimize, we use convex optimization to perform the following process:

(4) $$\begin{array}{c} \begin{array}{c} \min\;{\left\|A\right\|}_{0}\\ \text{s}.\text{t}.\;X=XA,\;A_{ii}=0. \end{array} \end{array}$$

However, the solution of the *l*_0_− norm is an NP-Hard problem in practical problems. Usually the *l*_1_− norm is used to replace the *l*_0_− norm to solve, so as to convert the subspace representation model to

(5) $$\begin{array}{c} \begin{array}{c} \min\;{\left\|A\right\|}_{1}\\ \text{s}.\text{t}.\;X=XA,\;A_{ii}=0. \end{array} \end{array}$$

### 3.3. Brain Tumor Image Segmentation Based on Sparse Subspace Clustering

**Figure 6:**
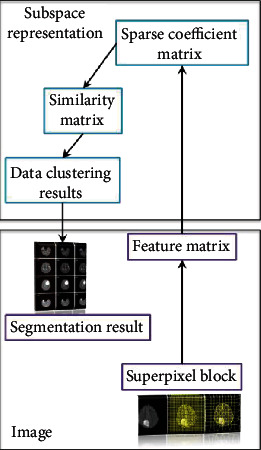
Image segmentation framework based on sparse subspace clustering.

Image segmentation is the process of segmenting images into nonoverlapping regions and extracting ROI from them, while sparse subspace clustering is a process used to cluster data of the same class into the same subspace. An image contains multiple target images with a complex texture structure, but the features on the image are composed of multiple low-dimensional subspace data. Therefore, the sparse subspace clustering algorithm can be used to segment the image. First, divide the image to be divided into multiple superpixel blocks, and divide the superpixel blocks of the same target image into the same subspace, so as to achieve the purpose of extracting the target image. The process is shown in Figure 6.

**Figure 7:**
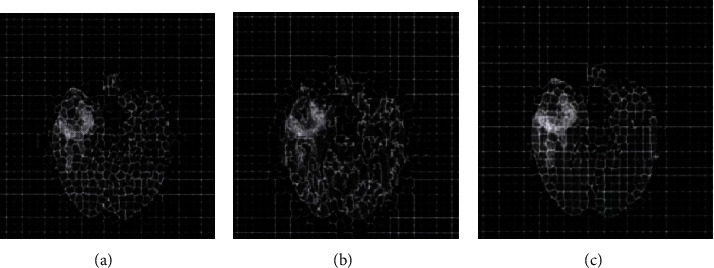
FLAIR image segmentation result when the *m* value changes. (a) Superpixel segmentation result when *m* = 10. (b) Superpixel segmentation result when *m* = 20. (c) Superpixel segmentation result when *m* = 30.

A variety of modal image fusion strategies use linear fusion. Linear fusion is the simplest multimodal MRI brain tumor image fusion method. It is a pixel-level fusion method, and the processing object is pixels. It is mainly to operate the pixel unit in each modal brain image, so as to comprehensively process the pixel information in each modal brain tumor image. Through the linear fusion operation, multimodal brain images can be converted into single-modal brain images containing multimodal brain tumor tissue information. Thus, multimodal image segmentation is converted into single-modal image segmentation, and the operation of multimodal processing is simplified. The specific operation of linear fusion is as follows:

(6) $$\begin{array}{c} F_{ij}=\alpha T_{1}\left(i,j\right)+\beta T_{2}\left(i,j\right)+\varepsilon T_{3}\left(i,j\right), \end{array}$$

where *F*_*ij*_ is the fused image; *T*_1_(*i*, *j*), *T*_2_(*i*, *j*), and *T*_3_(*i*, *j*) are the pixel values of *T*_1_, *T*_2_, and *T*_3_ at position (*i*, *j*); and *α*, *β*, and *ε* are the weights of each modal image, and meets *α* + *β* + *ε* = 1.  Figure 7 is a fusion image of multimodal images. Using the linear fusion operation, we use the following Flair ratio to obtain the fusion image in the figure: T1 : T1c : T2 = 3 : 2 : 1 : 4. After preprocessing the fused image, SSC can be used to complete the multimodal image segmentation.

The steps of the SSC-based multimodal image segmentation algorithm are as follows:

1. Input image *I* and use the preprocessing algorithm described in Section 3.1 to divide the fused image into *N* superpixel blocks
2. Extract *D*-dimensional feature vectors from superpixel blocks to form a feature matrix {*X*_*i*_} (*i* = 1, 2, ⋯, *n*)
3. Use the basic model of sparse subspace clustering to obtain the sparse coefficient matrix *C*
4. Calculate the similarity matrix *W* = |*C*| + |*C*^*T*^|, where*w*_*ij*_ = *w*_*ji*_ = |*c*_*ij*_| + |*c*_*ji*_|
5. The clustering result is obtained by using the spectral clustering algorithm

## 4. Simulation Experiment Analysis

### 4.1. Experiment-Related Settings

The comparison algorithms mainly include FCM, SVM, and the top 15 results of the Brats 2015 challenge. The experimental data of this paper is Brats 2015 [28]. The database contains data of two types of patients, those with benign tumors and those with malignant tumors, and contains brain image data of 274 patients. Each patient's brain image data contains Flair images, T1 images, T1c images, T2 images, and golden section results. The size of each modal image is 240∗240. We randomly selected data from 25 patients with brain tumors. Each patient's data includes five parts, namely, the Flair mode, the T1 mode, the T1c mode, the T2 mode, and the golden section results. The data size of each mode is 240∗240∗163. Because the two-dimensional tumor pictures of the same patient are similar, a set of two-dimensional multimodal brain tumor images is extracted from the data of each patient. There was a total of 25 sets of multimodal brain tumor image data. Among them, there are 15 groups of malignant tumor data and 10 groups of benign tumor data.

The performance of the algorithm in this paper mainly depends on the quality of the superpixels. The quality of the superpixels is controlled by the number *K* of the superpixels and the compact factor *m*. In this paper, the SLIC superpixel segmentation method needs to consider the density factor *m* and the number of target superpixel blocks [36]. In order to study the influence of the density factor *m*, the number*n*of predefined superpixel blocks is 1000 at first. Then, we explore the impact of the change of the compact factor size on the segmentation results. The compact factor*m* = 10 leads to a more rigid boundary, while *m* = 20 will produce a very flexible boundary, but it will increase the shape and irregularity of the superpixel.  Figure 7 is the result of FLAIR image segmentation when the value of *m* is different. By visually checking the superpixel boundary and area, when *m* = 20, the boundary can obtain a better segmentation result.

**Figure 8:**
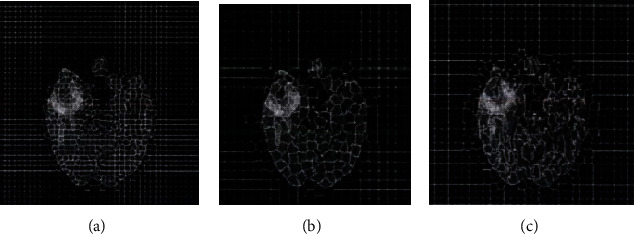
FLAIR image segmentation result when the *n* value changes. (a) Superpixel segmentation result when *n* = 300. (b) Superpixel segmentation result when *n* = 500. (c) Superpixel segmentation result when *n* = 1000.

The next step is to determine the number of target superpixel blocks.  Figure 8 shows the result of the FLAIR image segmentation when the value of *m* is 20 and the number *n* of the target superpixel blocks is different. When the compaction factor is fixed at *m* = 20, by changing the number*n*of the target superpixel blocks, the Dice measure is used to evaluate the formation performance of the superpixels.

Based on the above experimental results, the compact factor *m* = 20 in this experiment and the number of superpixels *n* = 500. The fuzzy factor in FCM is 2, and the parameter in SVM *σ* ∈ [10^−5^, 10^5^].

### 4.2. Evaluation Index

There are four evaluation indicators commonly used in objective evaluation criteria, namely, the Dice coefficient, the Jaccard coefficient, the false positive rate (Precision), and the true positive rate (Recall). The four evaluation indicators are shown in Table 1.

### 4.3. Simulation Results and Analysis

Table 2 shows the evaluation index results of the algorithm for different groups of multimodal image segmentation results, and Table 3 shows the top 15 segmentation results of the Brats 2015 challenge. It can be seen from the comparison of the data in the table that the average Dice index of this algorithm is 0.8577. Compared with the top 15 of the Brats 2015 competition, the accuracy is not much different, and it can even exceed the results of several of the rankings. The average Precision index is as high as 0.9615, which is a big advantage compared with the top 15 data. Compared with the top 15, the true positive rate is slightly inadequate. This is because the top 15 competition algorithms use a deep learning algorithm to segment the tumor in three dimensions and use the three-dimensional information of the brain tumor. Considering comprehensively, the algorithm in this paper can use the two-dimensional information of brain tumors to obtain a segmentation accuracy similar to the top 15 algorithms in the competition. It can be seen that the algorithm in this paper has certain value.

Table 4 shows the comparison of the average evaluation indexes of these three algorithms in 25 sets of data tests. From the comparison of the data in the table, we can see that the SSC algorithm used in each index is greatly improved compared to the other two algorithms.

**Figure 9:**
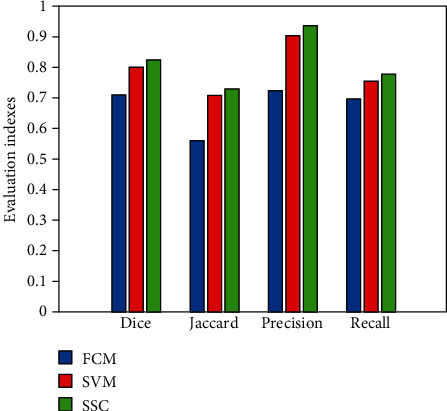
Comparison of evaluation indexes of various algorithms.

Figure 9 is a comparison of the histograms of the various evaluation methods on the four evaluation indicators. From the figure, the greater advantages of the SSC algorithm can be clearly found.

In order to verify the noise resistance of the SSC algorithm, this paper adds 5%, 10%, 15%, and 20% Gaussian noise to the original image. The segmentation results after noise addition are shown in Tables 5–8. From the changing trends of the values of the four evaluation indicators in Tables 5–8, it can be analyzed that the tumor segmentation effect decreases with increasing noise. The greater the noise content, the worse the segmentation effect. This is completely consistent with theory.

**Figure 10:**
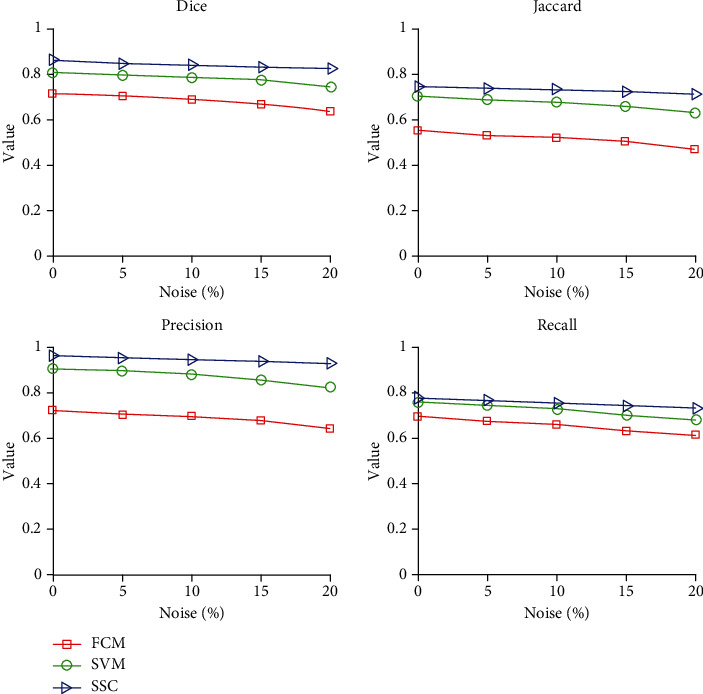
Segmentation performance of three algorithms under different noise contents.

Table 9 gives a comparison of the segmentation performance of the three algorithms under different noise ratios. Each data in the table is the average value of the above 25 sample data after division.  Figure 10 shows the changing trend of the segmentation accuracy of the three algorithms with increasing noise. It can be concluded from Table 9 and Figure 10 that the SSC algorithm is relatively better in terms of the antinoise performance of the three algorithms. As the amount of noise increases, the performance of FCM declines the fastest, followed by SVM, and the relative decline of SSC is smaller. This further illustrates the feasibility and reference value of the SSC algorithm selected in this paper.

## 5. Conclusion

Different features have different effects on tumor segmentation results. In order to make better use of multimodal brain tumor image information, this paper proposes an SSC-based multimodal brain tumor image segmentation method. First, linear fusion is used to fuse multiple single-modality brain MRI images into one image to be processed; secondly, superpixel features are extracted to construct a feature matrix; and finally, a sparse subspace clustering algorithm based on sparse representation is used to complete the segmentation. Using Brats 2015 competition data to experiment with the proposed method, the results show that the method used can well integrate the tumor information of the multimodal images and obtain good segmentation results. After adding different proportions of noise, the segmentation performance of the proposed algorithm decreases significantly slower than that of the comparison algorithm, which also verifies that the proposed algorithm has good noise resistance. However, the method used in this paper has certain limitations. It needs to optimize the weights of various modal data fusions, which is very time consuming.

## Figures and Tables

**Table 1:** Evaluation indicator introduction.

Number	Index	Explanation
1	Dice(*P*, *T*) = 2|*P*∩*T*|/|*P*| + |*T*|.	The Dice coefficient is a set similarity measurement method. In the image, it mainly refers to the degree to which the actual segmentation result and the golden segmentation result overlap each other, and the value is [0, 1]. Among them, 0 represents that there is no overlap between the actual segmentation result and the golden segmentation result, which represents the worst segmentation accuracy at this time, and 1 represents that the actual segmentation result and the golden segmentation result completely overlap, which represents the optimal segmentation accuracy at this time.
2	Jaccard(*P*, *T*) = |*P*∩*T*|/|*P*| + |*T*| − |*P*∩*T*|.	The Jaccard coefficient is a method similar to the Dice coefficient that relies on similarity as a measure. It describes the degree of overlap between the actual segmentation result and the golden segmentation result from another perspective.
3	Precision(*P*, *T*) = |*P*∩*T*|/|*P*|.	The false positive rate (Precision) reflects the accuracy of the actual segmentation result. The ratio of the overlap between the actual segmentation result and the golden segmentation result is used for description. The higher the ratio, the higher the proportion of the golden result included in the actual segmentation result.
4	Recall(*P*, *T*) = |*P*∩*T*|/|*T*|.	The true positive rate (Recall) reflects the accuracy of the actual results in the actual segmentation results. It refers to the ratio of the overlap between the actual and golden section results. The higher the ratio, the higher the proportion of the true segmentation result in the golden section.

**Table 2:** Comparison of multimodal image segmentation results.

Experimental sample		Index			
		Dice	Jaccard	Precision	Recall
Malignant tumor	1	0.8923	0.8042	0.9627	0.8287
	2	0.8596	0.7752	0.9748	0.7789
	3	0.8385	0.7431	0.9263	0.7821
	4	0.9507	0.8785	0.9874	0.9264
	5	0.9310	0.7886	0.9845	0.7954
	6	0.8746	0.7964	0.9678	0.8103
	7	0.9152	0.8522	0.9034	0.9371
	8	0.8386	0.7371	0.9976	0.7352
	9	0.8731	0.7796	0.9948	0.7911
	10	0.8694	0.7649	0.9563	0.8002
	11	0.8627	0.7628	0.9915	0.7832
	12	0.7016	0.5364	0.9997	0.5349
	13	0.8018	0.6742	0.9306	0.7132
	14	0.8220	0.6976	0.9736	0.7120
	15	0.8129	0.6842	0.9637	0.7058
Bright tumor	1	0.7961	0.6425	0.9264	0.6779
	2	0.8129	0.8413	0.9779	0.8646
	3	0.9298	0.6624	0.9836	0.6732
	4	0.9401	0.6830	0.7375	0.9118
	5	0.9228	0.8698	0.9990	0.8769
	6	0.9418	0.8891	0.9862	0.8996
	7	0.7147	0.5510	0.9996	0.5534
	8	0.7753	0.6256	0.9676	0.6394
	9	0.9027	0.8244	0.9834	0.8426
	10	0.8624	0.7632	0.9623	0.7824
Mean		0.8577	0.7451	0.9615	0.7743

**Table 3:** The top 15 segmentation results of the Brats 2015 challenge.

Rank	Dice	Precision	Recall
1	0.8730	0.8715	0.8916
2	0.8710	0.8621	0.9140
3	0.8720	0.8531	0.8633
4	0.8511	0.8619	0.8633
5	0.8739	0.8532	0.9180
6	0.8650	0.8530	0.9011
7	0.8325	0.8344	0.8457
8	0.8670	0.8623	0.8820
9	0.7760	0.7475	0.8635
10	0.8513	0.8248	0.9150
11	0.8417	0.8345	0.8917
12	0.8580	0.8716	0.8635
13	0.8512	0.8343	0.8916
14	0.8327	0.8527	0.8363
15	0.8328	0.8055	0.9090

**Table 4:** Comparison of evaluation indexes of different segmentation methods.

Methods	Evaluation index			
	Dice	Jaccard	Precision	Recall
FCM	0.7110	0.5564	0.7205	0.6975
SVM	0.8012	0.7056	0.9013	0.7558
SSC	0.8577	0.7451	0.9615	0.7743

**Table 5:** Comparison of multimodal image segmentation results with 5% noise.

Experimental sample		Index			
		Dice	Jaccard	Precision	Recall
Malignant tumor	1	0.8123	0.7758	0.9036	0.8080
	2	0.8016	0.7469	0.9229	0.7568
	3	0.8001	0.7154	0.9086	0.7735
	4	0.8462	0.8369	0.9321	0.8858
	5	0.8528	0.7427	0.9650	0.7804
	6	0.8134	0.7528	0.9487	0.7940
	7	0.8347	0.8274	0.8936	0.9166
	8	0.8006	0.7144	0.9376	0.7130
	9	0.8104	0.7423	0.9721	0.7668
	10	0.8110	0.7417	0.9325	0.7878
	11	0.8234	0.7326	0.9639	0.7626
	12	0.6841	0.5146	0.9688	0.5229
	13	0.7695	0.6155	0.9129	0.7007
	14	0.7996	0.6639	0.9639	0.7013
	15	0.8005	0.6582	0.9470	0.6982
Bright tumor	1	0.7486	0.6301	0.9003	0.6663
	2	0.7952	0.8204	0.9575	0.8452
	3	0.8985	0.6471	0.9588	0.6620
	4	0.8625	0.6598	0.7176	0.9031
	5	0.8563	0.8446	0.9425	0.8206
	6	0.9012	0.8396	0.9393	0.8759
	7	0.6852	0.5329	0.9579	0.5414
	8	0.7410	0.6012	0.9493	0.6225
	9	0.8863	0.8071	0.9389	0.8223
	10	0.8401	0.7540	0.9522	0.7639
Mean		0.8110	0.7167	0.9315	0.7557

**Table 6:** Comparison of multimodal image segmentation results with 10% noise.

Experimental sample		Index			
		Dice	Jaccard	Precision	Recall
Malignant tumor	1	0.7585	0.7147	0.8452	0.7581
	2	0.7662	0.7020	0.8967	0.7052
	3	0.7596	0.6996	0.8746	0.7196
	4	0.8008	0.7989	0.9003	0.8320
	5	0.8020	0.7011	0.9114	0.7404
	6	0.7642	0.7102	0.9095	0.7462
	7	0.8001	0.7834	0.8482	0.8346
	8	0.7779	0.6996	0.9063	0.6730
	9	0.7823	0.7032	0.9101	0.7162
	10	0.7863	0.7142	0.9011	0.7285
	11	0.7903	0.7020	0.9039	0.7126
	12	0.6523	0.5011	0.9008	0.5028
	13	0.7124	0.6031	0.8557	0.6896
	14	0.7210	0.6313	0.8932	0.6745
	15	0.7695	0.6220	0.8712	0.6512
Bright tumor	1	0.7103	0.6102	0.8103	0.6326
	2	0.7533	0.7945	0.8410	0.8071
	3	0.8120	0.6103	0.8124	0.6426
	4	0.8236	0.6120	0.6731	0.8426
	5	0.8022	0.8008	0.8526	0.7945
	6	0.8471	0.8106	0.8989	0.8142
	7	0.6326	0.5030	0.9009	0.5231
	8	0.7002	0.5936	0.8855	0.6005
	9	0.8308	0.7852	0.8797	0.8030
	10	0.8001	0.7262	0.8722	0.7103
Mean		0.7662	0.6853	0.8702	0.7142

**Table 7:** Comparison of multimodal image segmentation results with 15% noise.

Experimental sample		Index			
		Dice	Jaccard	Precision	Recall
Malignant tumor	1	0.7010	0.6235	0.7788	0.6963
	2	0.7121	0.6417	0.8293	0.6625
	3	0.7006	0.6582	0.8126	0.6741
	4	0.7259	0.7128	0.8253	0.6701
	5	0.7361	0.6733	0.8256	0.6693
	6	0.7140	0.6682	0.7896	0.6642
	7	0.7263	0.7117	0.7526	0.6723
	8	0.7030	0.6336	0.7864	0.6008
	9	0.7234	0.6402	0.8124	0.6037
	10	0.7026	0.6513	0.8102	0.6395
	11	0.7263	0.6412	0.8006	0.6279
	12	0.6136	0.4562	0.8152	0.4963
	13	0.6742	0.5846	0.7852	0.6230
	14	0.6892	0.6006	0.7984	0.6172
	15	0.7211	0.6001	0.8010	0.6003
Bright tumor	1	0.6982	0.5895	0.7142	0.6110
	2	0.7120	0.7312	0.7265	0.6753
	3	0.7361	0.5742	0.7416	0.5996
	4	0.7216	0.5863	0.6246	0.7582
	5	0.7121	0.7323	0.7693	0.7296
	6	0.7132	0.7125	0.8263	0.7369
	7	0.6030	0.4852	0.8082	0.5020
	8	0.6482	0.5611	0.8060	0.5801
	9	0.7413	0.7230	0.8132	0.6778
	10	0.7143	0.6736	0.8007	0.6256
Mean		0.7028	0.6346	0.7862	0.6405

**Table 8:** Comparison of multimodal image segmentation results with 20% noise.

Experimental sample		Index			
		Dice	Jaccard	Precision	Recall
Malignant tumor	1	0.6013	0.5582	0.5786	0.6030
	2	0.6230	0.5631	0.6023	0.5436
	3	0.6058	0.5477	0.6113	0.5633
	4	0.6003	0.5693	0.6037	0.5721
	5	0.6146	0.5746	0.6012	0.5126
	6	0.6008	0.5832	0.5963	0.5362
	7	0.6001	0.5746	0.5748	0.5284
	8	0.6200	0.5365	0.5836	0.5369
	9	0.5963	0.5208	0.5862	0.5623
	10	0.5982	0.5300	0.5916	0.5123
	11	0.5996	0.5613	0.5746	0.5023
	12	0.5342	0.4203	0.5842	0.4523
	13	0.5846	0.5110	0.5532	0.5203
	14	0.5768	0.5030	0.5631	0.5417
	15	0.5636	0.5007	0.5711	0.5731
Bright tumor	1	0.5875	0.5114	0.5369	0.5324
	2	0.5939	0.5630	0.5284	0.6064
	3	0.5742	0.5023	0.5748	0.5412
	4	0.5936	0.5431	0.5303	0.6234
	5	0.5768	0.5623	0.5923	0.6127
	6	0.5693	0.5665	0.5830	0.6471
	7	0.5234	0.4528	0.5746	0.4864
	8	0.5236	0.5220	0.5822	0.5520
	9	0.5741	0.5360	0.5623	0.6113
	10	0.5698	0.5142	0.5722	0.5436
Mean		0.5842	0.5331	0.5765	0.5527

**Table 9:** Comparison of segmentation performance of three algorithms under different noise ratios.

Noise ratio	Algorithm	Dice	Jaccard	Precision	Recall
5%	FCM	0.7002	0.5316	0.7010	0.6753
	SVM	0.7912	0.6902	0.8956	0.7412
	SSC	0.8410	0.7367	0.9515	0.7657
10%	FCM	0.6833	0.5241	0.6931	0.6595
	SVM	0.7800	0.6789	0.8763	0.7286
	SSC	0.8362	0.7353	0.9402	0.7542
15%	FCM	0.6658	0.5056	0.6767	0.6323
	SVM	0.7682	0.6574	0.8553	0.7001
	SSC	0.8268	0.7246	0.9362	0.7405
20%	FCM	0.6312	0.4712	0.6420	0.6125
	SVM	0.7404	0.6310	0.8211	0.6803
	SSC	0.8182	0.7131	0.9265	0.7327

## Data Availability

The labeled dataset used to support the findings of this study are available from the corresponding author upon request.

## References

[1] Siegel R., Naishadham D., Jemal A. (2013). Cancer statistics, 2013. *CA: a Cancer Journal for Clinicians*.

[2] Gordillo N., Montseny E., Sobrevilla P. (2013). State of the art survey on MRI brain tumor segmentation. *Magnetic Resonance Imaging*.

[3] Gibbs P., Buckley D. L., Blackband S. J., Horsman A. (1996). Tumour volume determination from MR images by morphological segmentation. *Physics in Medicine and Biology*.

[4] Stadlbauer A., Moser E., Gruber S. (2004). Improved delineation of brain tumors: an automated method for segmentation based on pathologic changes of 1H-MRSI metabolites in gliomas. *NeuroImage*.

[5] Shanthi K. J., Kumar M. S. Skull stripping and automatic segmentation of brain MRI using seed growth and threshold techniques.

[6] Kaus M. R., Warfield S. K., Nabavi A., Black P. M., Jolesz F. A., Kikinis R. (2001). Automated segmentation of MR images of brain tumors. *Radiology*.

[7] Lakare S., Kaufman A. (2000). 3D segmentation techniques for medical volumes. *Center for Visual Computing, Department of Computer Science*.

[8] Letteboer M., Niessen W., Willems P., Dam E. B., Viergever M. Interactive multi-scale watershed segmentation of tumors in MR brain images.

[9] Dam E., Loog M., Letteboer M. Integrating automatic and interactive brain tumor segmentation.

[10] Cates J. E., Whitaker R. T., Jones G. M. (2005). Case study: an evaluation of user-assisted hierarchical watershed segmentation. *Medical Image Analysis*.

[11] Clark M. C., Hall L. O., Goldgof D. B., Velthuizen R., Murtagh F. R., Silbiger M. S. (1998). Automatic tumor segmentation using knowledge-based techniques. *IEEE Transactions on Medical Imaging*.

[12] Capelle A. S., Alata O., Fernandez C., Lefevre S., Ferrie J. C. Unsupervised segmentation for automatic detection of brain tumors in MRI.

[13] Qian P., Chen Y., Kuo J.-W. (2020). mDixon-based synthetic CT generation for PET attenuation correction on abdomen and pelvis jointly using transfer fuzzy clustering and active learning-based classification. *IEEE Transactions on Medical Imaging*.

[14] Qian P., Jiang Y., Deng Z. (2016). Cluster prototypes and fuzzy memberships jointly leveraged cross-domain maximum entropy clustering. *IEEE Transactions on Cybernetics*.

[15] Yegnanarayana B. (2009). *Artificial Neural Networks*.

[16] Liu J., Jung H. W., Dubra A., Tam J. (2018). Cone photoreceptor cell segmentation and diameter measurement on adaptive optics images using circularly constrained active contour model. *Investigative Ophthalmology & Visual Science*.

[17] Gibou F., Fedkiw R., Osher S. (2018). A review of level-set methods and some recent applications. *Journal of Computational Physics*.

[18] Mahalakshmi, Krishnappa H. K., Jayadevappa D. (2019). Automated brain tumor segmentation and identification using MR images. *International Journal of Innovative Technology and Exploring Engineering*.

[19] Fischl B., Salat D. H., Busa E. (2002). Whole brain segmentation: automated labeling of neuroanatomical structure in the human brain. *Neuron*.

[20] Pohl K. M., Fisher J., Levitt J. J., Duncan J. S., Gerig G. (2005). A unifying approach to registration, segmentation, and intensity correction. *Medical Image Computing and Computer-Assisted Intervention – MICCAI 2005*.

[21] Prastawa M., Bullitt E., Ho S., Gerig G. (2004). A brain tumour segmentation framework based on outlier detection. *Medical Image Analysis*.

[22] Gering D. T., Grimson W. E. L., Kikinis R., Dohi T., Kikinis R. (2002). Recognizing deviations from normalcy for brain tumor segmentation. *Medical Image Computing and Computer-Assisted Intervention — MICCAI 2002*.

[23] Cuadra M. B., Pollo C., Bardera A., Cuisenaire O., Villemure J. G., Thiran J. P. (2004). Atlas-based segmentation of pathological MR brain images using a model of lesion growth. *IEEE Transactions on Medical Imaging*.

[24] Gooya A., Pohl K. M., Bilello M. (2012). GLISTR: glioma image segmentation and registration. *IEEE Transactions on Medical Imaging*.

[25] Cobzas D., Birkbeck N., Schmidt M., Jagersand M., Murtha A. 3D variational brain tumor segmentation using a high dimensional feature set.

[26] Ho S., Bullitt E., Gerig G. Level-set evolution with region competition: automatic 3-D segmentation of brain tumors.

[27] Lee C. H., Wang S., Murtha A., Brown M. R. G., Greiner R., Metaxas D., Axel L., Fichtinger G., Székely G. (2008). Segmenting brain tumors using pseudo-conditional random fields. *Medical Image Computing and Computer-Assisted Intervention – MICCAI 2008*.

[28] Geremia E., Clatz O., Menze B. H., Konukoglu E., Criminisi A., Ayache N. (2011). Spatial decision forests for MS lesion segmentation in multi-channel magnetic resonance images. *Neuro Image*.

[29] Zikic D., Glocker B., Konukoglu E., Ayache N., Delingette H., Golland P., Mori K. (2012). Decision forests for tissue-specific segmentation of high grade gliomas in multi-channel MR. *Medical Image Computing and Computer-Assisted Intervention – MICCAI 2012*.

[30] Zikic D., Glocker B., Konukoglu E. Context-sensitive classification forests for segmentation of brain tumor tissues.

[31] Menze B. H., Geremia E., Ayache N., Szekely G. Segmenting glioma in multi-modal images using a generative model for brain lesion segmentation.

[32] Menze B. H., Jakab A., Bauer S. (2015). The multimodal brain tumor image segmentation benchmark (BRATS). *IEEE Transactions on Medical Imaging*.

[33] Bauer S., Wiest R., Nolte L. P., Reyes M. (2013). A survey of MRI-based medical image analysis for brain tumor studies. *Physics in Medicine & Biology*.

[34] Ehsan E., René V. (2013). Sparse subspace clustering: algorithm, theory, and applications. *IEEE Transactions on Pattern Analysis and Machine Intelligence*.

[35] Angulakshmi M., Lakshmi Priya G. G. (2019). Walsh Hadamard transform for simple linear iterative clustering (SLIC) superpixel based spectral clustering of multimodal MRI brain tumor segmentation. *IRBM*.

[36] Radhakrishna A., Appu S., Kevin S., Aurélien L., Pascal F., Sabine S. (2010). *SLIC superpixels*.

